# Comparison of endoscopic submucosal resection with ligation and endoscopic submucosal dissection for small rectal neuroendocrine tumors: A multicenter retrospective study

**DOI:** 10.1002/deo2.163

**Published:** 2022-09-15

**Authors:** Kenshi Matsuno, Hideaki Miyamoto, Hideki Kitada, Shinichi Yoshimatsu, Fumio Tamura, Kouichi Sakurai, Kotaro Fukubayashi, Takashi Shono, Hiroko Setoyama, Taichi Matsuyama, Shinichiro Suko, Rei Narita, Munenori Honda, Masakuni Tateyama, Hideaki Naoe, Jun Morinaga, Yasuhito Tanaka, Ryosuke Gushima

**Affiliations:** ^1^ Department of Gastroenterology and Hepatology Faculty of Life Sciences Kumamoto University Kumamoto Japan; ^2^ Department of Gastroenterology Japanese Red Cross Kumamoto Hospital Kumamoto Japan; ^3^ Department of Gastroenterology Kumamoto General Hospital Community Health Care Organization Kumamoto Japan; ^4^ Department of Gastroenterology Kumamoto Regional Medical Center Kumamoto Japan; ^5^ Hattori Clinic Kumamoto Japan; ^6^ Department of Gastroenterology Tamana Central Hospital Kumamoto Japan; ^7^ Department of Gastroenterology Kumamoto Chuo Hospital Kumamoto Japan; ^8^ Department of Gastroenterology Kumamoto Rosai Hospital Kumamoto Japan; ^9^ Department of Gastroenterology National Hospital Organization Kumamoto Medical Center Kumamoto Japan; ^10^ Department of Gastroenterology Saiseikai Kumamoto Hospital Kumamoto Japan; ^11^ Department of Gastroenterology Minamata City Hospital and Medical Center Kumamoto Japan; ^12^ Department of Clinical Investigation (Biostatistics) Kumamoto University Hospital Kumamoto Japan

**Keywords:** colonoscopy, endoscopic resection, endoscopic submucosal dissection, endoscopic submucosal resection with band ligation, rectal neuroendocrine tumors

## Abstract

**Objectives:**

Endoscopic submucosal resection with band ligation (ESMR‐L) and endoscopic submucosal dissection (ESD) are both standard endoscopic resection methods for rectal neuroendocrine tumors (NETs) <10 mm in size. However, there is no definitive consensus on which is better. Here, we compared the efficacy of ESMR‐L and ESD for small rectal NETs.

**Methods:**

This was a multicenter retrospective cohort study including 205 patients with rectal NETs who underwent ESMR‐L or ESD. Treatment outcomes were compared by univariate analysis, multivariate analysis, and inverse probability treatment weighting (IPTW) using propensity scores. Subgroup analysis evaluated the impact of the endoscopist's experience on the technical outcome.

**Results:**

Eighty‐nine patients were treated by ESMR‐L and 116 by ESD. The R0 resection rate was not significantly different between the two (90% vs. 92%, *p* = 0.73). The procedure time of ESMR‐L was significantly shorter than for ESD (17 min vs. 52 min, *p* < 0.01) and the hospitalization period was also significantly shorter (3 days vs. 5 days, *p* < 0.01). These results were confirmed by multivariate analysis and also after IPTW adjustment. The procedure time of ESD was significantly prolonged by a less‐experienced endoscopist (49 min vs. 70 min, *p* = 0.02), but that of ESMR‐L was not affected (17 min vs. 17 min, *p* = 0.27).

**Conclusions:**

For small rectal NETs, both ESMR‐L and ESD showed similar high complete resection rates. However, considering the shorter procedure time and shorter hospitalization period, ESMR‐L is the more efficient treatment method, especially for less‐experienced endoscopists.

## INTRODUCTION

Rectal neuroendocrine tumors (NETs) are one of the most common types of digestive NETs. The incidence of detected rectal NETs has increased in the past few decades due to the increased number of colonoscopies.^1^, ^2^, ^3^, ^4^, ^5^ As rectal NETs <10 mm in size carry a low risk of lymph node metastasis,^6^, ^7^, ^8^, ^9^ current guidelines recommend local excision including endoscopic resection (ER) for these tumors.^10^

There are many established ER methods, including conventional endoscopic mucosal resection (c‐EMR),^11^ endoscopic submucosal resection with band ligation (ESMR‐L),^12^ endoscopic submucosal resection with cap aspiration (ESMR‐C),^13^ EMR after circumferential incision (precutting EMR),^14^ and endoscopic submucosal dissection (ESD).^15^ Of these, especially ESMR‐L and ESD are commonly performed procedures for rectal NET resection in daily clinical practice. Several reports address the efficacy of modified EMR (ESMR‐L, ESMR‐C, and precutting EMR) and ESD.^16^, ^17^, ^18^, ^19^ However, most of these reports are from a single high‐volume center, and there are a few from multiple facilities. Furthermore, there have been no reports focused on the impact of the endoscopist's experience on treatment outcomes for each of these ER methods. Thus, additional evidence is required to establish an optimal strategy for ER in rectal NETs. Therefore, we surveyed the results of ER treatments for small rectal NETs in a multi‐center database, focusing on a comparison between ESMR‐L and ESD.

## METHODS

### Patients

This was a multicenter retrospective cohort study of patients who underwent ESMR‐L or ESD from 2010 to 2019 for rectal NETs <10 mm in size. This study was conducted at 11 Japanese facilities, including university hospitals, general hospitals, and practicing clinics. Relevant data of the patients and lesions collected from the clinical records were analyzed. The evaluation items were as follows: clinical background of the patient, the ER method, the procedure time, perforations and postoperative bleeding as complications, the R0 resection rate, and the hospitalization period. Patients who were treated other than by ESMR‐L or ESD, such as by c‐EMR, ESMR‐C, or precutting EMR, were excluded. Patients with multiple lesions were also excluded.

Written informed consent was obtained from all patients prior to treatment. This study was approved by the Ethical Committee of Kumamoto University Hospital (approval number 2015) and performed in accordance with the ethical principles associated with the Declaration of Helsinki.

### Endoscopic procedures

In accordance with the guidelines, rectal NETs less than 10 mm without obvious muscular invasion or metastasis were indicated for ER. Most cases were treated after the histological diagnosis of NET by endoscopic biopsies. Prior to the procedure, endoscopic ultrasonography to rule out muscle layer invasion and computed tomography to rule out metastases were usually performed.

In the ESMR‐L procedure, the lesion is aspirated and ligated using a ligation device (Sumitomo Bakelite Co. Ltd., Tokyo, Japan) after submucosal injection, and resected with a snare placed below the ligation band. Routinely, saline was used for submucosal injection, and endoscopic closure of mucosal defects was with clips after resection.

In the ESD procedure, incision and deeper cutting create a mucosal flap after submucosal injection; thereafter, a circumferential incision with submucosal dissection is performed until the tumor can be completely removed. A Dual knife (KD‐650Q; Olympus) or a Flush knife (DK2618JB; Fujifilm) was mainly used for the ESD procedure. Coagulation was occasionally performed with hemostatic forceps for the exposed vessels after resection. Routinely, 0.4% hyaluronic acid (MucoUP; Boston Scientific Japan Co.) was used, and endoscopic closure for mucosal defects was not conducted after resection.

All of the procedures were carried out by a total of 60 different endoscopists. The choice of ER method was made by each endoscopist.

### Histological evaluation

Hematoxylin‐Eosin and immunohistochemical staining (chromogranin A, synaptophysin) was performed on formalin‐fixed, paraffin‐embedded samples after ER. Tumor size, invasion depth, lymphatic and vascular involvement, and tumor involvement in the lateral and vertical margins, were histologically assessed. The lympho‐vascular invasion was usually evaluated by immunostaining (CD34, Victoria blue, and D2–40). Ki‐67 was used to evaluate cell proliferation and classify the grade of the tumor. Grade 1 was defined as having a mitotic count <2 per 10 high‐power fields and/or Ki67 ≤2%. The pathological diagnosis was based on the 2010 World Health Organization classification of tumors of the digestive system.

### Definition

The procedure time was defined as the time from insertion to the removal of the endoscope. Perforation was defined as a visible hole in the rectum wall recognized during the procedure, or free air detected by abdominal computed tomography after the procedure. Postoperative bleeding was defined as the presence of fresh bloody stool, which required urgent endoscopy for hemostasis. R0 resection was defined as en bloc resection with tumor‐free lateral and vertical margins. “Experienced” endoscopists were defined as having performed more than 3000 colonoscopies and 500 colonic c‐EMR and/or 30 colonic ESD at each point of treatment.

### Statistical analysis

Clinical outcomes were analyzed using the Mann‐Whitney U test, Student's *t*‐test, Fisher's exact test, or chi‐squared test, as appropriate. Multivariate logistic and linear regression models were also employed based on propensity scores to adjust for candidate confounding factors. To generate the propensity score, logistic regression was performed using the ER method as the outcome, and the five variables were age, sex, tumor location, tumor size, and endoscopists’ experience. Linear regression models using patients from each medical facility as a cluster were employed to evaluate associations between treatment and outcome. In addition, the inverse probability of treatment weighting (IPTW) method based on propensity scoring was used to estimate the average treatment effect of ESMR‐L compared to ESD. In these models, outcome variables such as ESD procedure time or dissection speed were log‐transformed because of their skewed distribution. Thus, in the multivariate linear regression models, the ESMR‐L and ESD procedure times were compared by estimating the exponentials of regression coefficients. Probability values for statistical tests were two‐tailed and *p* < 0.05 was considered significant. Statistical analysis was performed with R version 3.3.3 (R Foundation, Vienna, Austria), and STATA15.1 (StataCorp, Lakeway Drive, Texas).

## RESULTS

**FIGURE 1:**
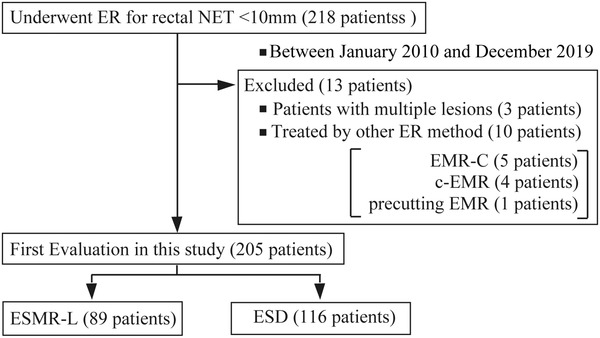
Study flow diagram

From January 2010 to December 2019, a total of 218 patients who underwent ER for rectal NETs <10 mm enrolled from 11 institutions for this study. Of these, we excluded 10 patients who underwent ER by a method other than ESMR‐L or ESD (these were four c‐EMR, five ESMR‐C, and one precutting EMR). We also excluded three patients who had multiple NET lesions resected in a single procedure. Hence, we finally analyzed 205 patients, 89 of whom were treated by ESMR‐L, and 116 by ESD. The study flowchart is shown in Figure 1.

The characteristics of the patients and their lesions are shown in Table 1. The lesions were mainly located in the lower rectum (*n* = 177, 86%), and the median tumor size was 5 mm. The mean age of the patients who underwent ESD was higher than that of ESMR‐L (53±13 years vs. 58 ± 13 years, *p* = 0.02), and the median tumor size in the former was larger (4 mm vs. 5 mm, *p* < 0.01). The proportion of experienced endoscopists was significantly greater than for ESD compared to ESMR‐L (80% vs. 55%, *p* = 0.04).

**TABLE 1:** Clinicopathological characteristics of the patients and lesions

**Group**	**Total**	**ESMR‐L**	**ESD**	***p*‐value**
No. of patients	205	89	116	
Age, mean ± SD, years	56 ± 13.2	53 ± 13	58 ± 13	0.02
Sex, male, *n* (%)	111 (54)	48 (54)	63 (54)	>0.99
Tumor location, *n* (%)				0.14
Rb/Ra/Rs	177 (86)/26/2	74 (83)/15/0	103 (89)/11/2	
Tumor size, median (IQR), mm	5 (3–6)	4 (3–5)	5 (4–7)	<0.01
Operator, *n* (%)				0.04
Experienced/less‐experienced	152 (74)/53	59 (67)/30	93 (80)/23	
Lympho‐vascular invasion, *n* (%)	27 (13)	16 (18)	11 (10)	0.10
WHO grade, G1/G2, n. (%)	198 (97)/7	85 (96)/4	113 (97)/3	0.47

Abbreviations: ESD, endoscopic submucosal dissection; ESMR‐L, endoscopic submucosal resection with ligation; IQR, interquartile range; Ra, lower rectum; Rb, lower rectum; Rs, rectosigmoid; SD, standard deviation; WHO, World Health Organization.

Table 2 depicts the univariate analysis of treatment outcomes. All lesions were resected en bloc either by ESMR‐L or ESD, with no significant difference in the R0 resection rate (90% vs. 92%, *p* = 0.73). The procedure time was significantly shorter for ESMR‐L than for ESD patients (17 min vs. 52 min, *p* < 0.01), and the hospitalization period was also shorter for ESMR‐L patients (3 days vs. 5 days, *p*< 0.01). No instance of perforation was found in any of the patients, and the rate of postoperative bleeding was not significantly different (7% vs. 2%, *p* = 0.14). All cases of postoperative bleeding were successfully managed with endoscopic hemostasis, and no blood transfusion or surgery was required. We also showed the clinical outcomes of each 11 institutions in Table S1.

**TABLE 2:** Technical results and complications

**Method**	**ESMR‐L**	**ESD**	
	***n* = 89**	***n* = 116**	***p*‐value**
Procedure time, median (IQR), min.	17 (12–23)	52 (33–78)	<0.01
En bloc resection rate, %	100	100	
R0 resection rate, %	90	92	0.73
Histological margin			
HM, 0/1/X, no. (%)	86 (97)/0/3	116 (100)/0/0	0.08
VM, 0/1/X, no. (%)	81 (91)/3/5	107 (92)/7/2	0.80
Both involvement, no. (%)	2 (2)	0 (0)	0.19
Complications, no. (%)			
Perforation	0 (0)	0 (0)	
Delayed bleeding	6 (7)	2 (2)	0.14
Hospitalization period, median (IQR), day.	3 (1–4)	5 (4–9)	<0.01

Abbreviations: ESD, endoscopic submucosal dissection; ESMR‐L, endoscopic submucosal resection with ligation; HM, horizontal margin; IQR, interquartile range; RVM, vertical margin.

Multivariate regression analysis was then applied to compare ESMR‐L with ESD, confirming that the procedure time was significantly shorter for the former (odds ratio = 0.32, 95% confidence interval [CI] 0.26–0.40, *p* < 0.001) and that the R0 resection rate was not significantly different (odds ratio = 0.62, 95% CI 0.08–4.58, *p* = 0.641; Table 3). The hospitalization period was also confirmed to be significantly shorter after ESMR‐L (odds ratio = 0.45, 95% CI 0.24–0.86, *p* = 0.021; Table 3). In the IPTW analysis, the estimated mean procedure time when using the ESMR‐L method was also significantly shorter than for ESD (odds ratio = 0.33, 95% CI 0.27–0.41, *p* < 0.001), and again, the R0 resection rate was not significantly different (odds ratio = 0.98, 95% CI 0.89–1.07, *p* = 0.614; Table 3). The hospitalization period was also significantly shorter for patients after ESMR‐L (odds ratio = 0.46, 95% CI 0.39–0.53, *p* < 0.001; Table 3).

**TABLE 3:** Technical outcomes for endoscopic submucosal resection with ligation compared with endoscopic submucosal dissection (ESD) by univariate, multivariate, and inverse probability of treatment weighting method using propensity score analysis

	**Crude**		**Multivariate**		**IPTW method**	
	**Odds ratio**		**Odds ratio**		**Odds ratio**	
	**(95% CI)**	***p*‐value**	**(95% CI)**	***p*‐value**	**(95% CI)**	***p*‐value**
Procedure time	0.33	<0.001	0.32	<0.001	0.33	<0.001
	(0.27–0.41)		(0.26–0.40)		(0.29–0.38)	
R0 resection rate	0.75	0.744	0.62	0.641	0.98	0.614
	(0.13–4.30)		(0.08–4.83)		(0.89–1.07)	
Hospitalization period	0.41	0.023	0.45	0.021	0.46	<0.001
	(0.23–0.86)		(0.24–0.86)		(0.98–0.53)	

Abbreviations: ESD, endoscopic submucosal dissection; ESMR‐L, endoscopic submucosal resection with ligation; IPTW, inverse probability of treatment weighting; Odds ratio, the outcome (procedure time, R0 resection rate, and hospitalization period) of ESMR‐L compared with ESD.

In the subgroup analysis, we evaluated the impact of the endoscopist's experience on the technical outcomes for each ER method. For the ESMR‐L, there was no difference in procedure time between experienced and less‐experienced endoscopists (17 min vs. 17 min, *p* = 0.269; Table 4). On the other hand, for ESD, the median procedure time was longer in cases treated by less‐experienced endoscopists (49 min vs. 70 min, *p* = 0.017; Table 4). There was no significant difference in the R0 resection rate whether performed by experienced or less‐experienced endoscopist both in ESMR‐L (86.4% vs. 96.7%, *p* = 0.263; Table 4) and ESD procedure (92.5% vs. 91.3%, *p* > 0.99; Table 4). The hospitalization period was longer in both the ESMR‐L (2 days vs. 3 days, *p* < 0.023; Table 4) and ESD groups (5 days vs. 8 days, *p* < 0.001; Table 4) when treated by less‐experienced endoscopists.

**TABLE 4:** Subgroup analysis to evaluate the impact of less‐experienced endoscopists on treatment outcomes

	**ESMR‐L(*n* = 89)**			**ESD(*n* = 116)**		
	**Experienced**	**Less‐experienced**		**Experienced**	**Less‐experienced**	
**ER method Endoscopist**	**(*n* = 59)**	**(*n* = 30)**	***p*‐value**	**(*n* = 93)**	**(*n* = 23)**	***p*‐value**
Procedure time, median (IQR), min.	17 (12–21)	17 (13–25)	0.269	49 (30–72)	70 (49–95)	0.0172
R0 resection rate (%)	86.4	96.7	0.263	92.5	91.3	>0.99
Hospitalization period, median (IQR), day.	2 (1–3)	3 (2–4)	0.024	5 (4–8)	8 (6–10)	<0.001

Abbreviations: ESD, endoscopic submucosal dissection; ESMR‐L, endoscopic submucosal resection with ligation; IQR, interquartile range.

We also examined the impact of endoscopist experience level on treatment outcomes using linear or logistic regression models adjusted by propensity score. The procedure time of ESD was significantly prolonged by less‐experienced endoscopists (odds ratio = 1.42, 95% CI 1.12–1.80, *p* = 0.009; Table S2), but that of ESMR‐L was not affected (odds ratio = 1.14, 95% CI 0.92–1.40, *p* = 0.205; Table S2). ESMR‐L performed by less‐experienced endoscopists did not result in lower R0 resection rates (odds ratio = 3.94, 95% CI 0.32–47.96, *p* = 0.282; Table S2), and that was also confirmed by the ESD procedure (odds ratio = 0.75, 95% CI 0.16–3.53, *p* = 0.716; Table S2).

In this study, 27 patients (13%) had a lympho‐vascular invasion. Of which, 13 underwent additional surgery, and one of them was found to have lymph node metastasis. No apparent lymph node recurrence was observed in patients who did not undergo additional surgery. There were no confirmed cases of local recurrence during the follow‐up period, with a median 35‐month (interquartile range 25%–75%, 14–61) follow‐up observation. In contrast, a distant metastatic recurrence was confirmed in one case in the ESD group. The lesion was NET grade 2 of WHO classification, 7 mm in size without lympho‐vascular invasion, and the horizontal and vertical margins were free. However, the patient did not accept the additional surgery. After more than 9 years passed since the ESD procedure, multiple liver masses were pointed out, and the biopsy revealed a recurrence of NET.

## DISCUSSION

In this retrospective study for rectal NETs <10 mm in size, the patients who underwent ESMR‐L required a significantly shorter procedure and shorter hospitalization period than those who underwent ESD, but there was no difference in the R0 resection rate. These results were confirmed after adjusting for confounding factors using both multivariate analysis and the IPTW method. Interestingly, the level of endoscopists’ experience significantly affected the procedure time for ESD more than for ESMR‐L, and ESMR‐L performed by less‐experienced endoscopists did not result in lower R0 resection rates. Both resection methods resulted in a longer hospitalization period when treated by less‐experienced endoscopists. There was no significant difference in the complication rates by endoscopist's experience. This might be because the patients treated by less‐experienced endoscopists had a longer hospital stay after treatment and were followed more carefully.

In our study, the R0 resection rate was not significantly different between ESMR‐L and ESD (90% vs. 92%, *p* = 0.73), similar to what has been previously reported (81%–100%, 54%–100%, respectively).^12^, ^13^, ^14^, ^15^, ^16^, ^17^, ^18^, ^19^, ^20^, ^21^, ^22^, ^23^, ^24^, ^25^ However, our simulation using the data of this study revealed that the statistical power to verify the non‐inferiority of ESMR‐L compared to ESD was low (<0.1), suggesting the necessity of further investigations with a large sample size. There have been two meta‐analyses comparing ESD and modified EMR, which include ESMR‐L and EMR‐C, as ER methods for rectal NETs.^26^, ^27^ The latest meta‐analysis showed no significant difference in the R0 resection rate between ESMR‐L and ESD, consistent with our results. All of these previous reports found that the procedure time of ESMR‐L was significantly shorter than ESD, as we also found. Most of these reports were from a single high‐volume center, and the number of cases in each report was small. Hence, in contrast, our study had a relatively large number of cases from multiple institutions, including the university hospital, general hospitals, and practicing clinics. The guidelines did not specify which resection method is recommended.^10^, ^28^ Additional evidence is still needed.

In most cases for which R0 resection was not achieved, the vertical margins were either unclear or positive. Free vertical margins are the most important for achieving the complete resection of small rectal NETS. For the ESD procedure, the submucosal dissection should be performed just above the muscular layer in order to avoid the burning effect on the tumor. However, this procedure benefits from more experienced endoscopic skills and entails a longer procedure time. In contrast, ESMR‐L is a relatively simple and rapid procedure not requiring so much endoscopic experience and skill. For this reason, the endoscopist's experience significantly affected the procedure time of ESD more than ESMR‐L. ESMR‐L also has an economic advantage because it does not require the use of various different endoscopic knives, hemostat forceps, and expensive liquids for submucosal injection. In addition, a shorter hospitalization period is favorable in terms of medical costs.

All of the horizontal margins were negative in the ESD group, whereas three cases of horizontal margins were unclear in the ESMR‐L group. This may be due to the fact that specimens after ESMR‐L were often not stretched and fixed on the board before being fixed in formalin. Although no additional surgical resection was performed in these three cases with unclear horizontal margins, none of them had obvious local recurrence. Of the three patients with unclear horizontal margins, two also had unclear vertical margins. Only one patient had unclear horizontal margins with free vertical margins. This lesion was located near the scar of c‐EMR that had been performed previously. ESD would be better indicated for such a lesion near the scar because of its flexibility in the resection area. ESD would also be more suitable for large rectal NET lesions, although it is not usually indicated for ER.

There is no definitive consensus on the definition of “experienced” endoscopists. In the systematic literature review of learning curves for colorectal polyp resection techniques,^29^ higher en bloc and complete resection rates were achieved in 50–300 cases with the colonic c‐EMR procedure and 20–40 cases with the ESD procedure. Furthermore, it was reported that the procedural speed was increased after 30 cases in the ESD procedure. Because only a few endoscopists had extensive experience with ESMR‐L procedures and colorectal ESD procedures in our study, we also defined “experienced” endoscopists with extensive colonoscopy and colonic c‐EMR experiences.

Our study has some limitations. First, this is a retrospective analysis of procedures. The choice of ER methods was dependent on the institution and the operator, and there were no clear criteria. Although we have adjusted for candidate confounding factors, it might be a major selection bias. Second, the timing of treatment might affect the technical results since the duration of our study period was 10 years. Third, because this study focused on treatment outcomes, the long‐term outcome data were insufficient to draw any conclusions in later clinical courses. Forth, the procedure time of our study seemed to be longer than in the previous reports. It might be due to the definition of procedure time as the time from insertion to the removal of the endoscope. Despite these limitations, the strength of our study is that it examined the impact of endoscopists´ experience on each ER method for rectal NETs with a relatively large number of cases, and analyzed the treatment outcome of many endoscopists a further strength.

In conclusion, compared with ESD, ESMR‐L had a shorter procedure time and shorter hospitalization period despite the similar R0 resection rate and complication rate. The procedure time of ESD was affected by the endoscopists’ experience but that of ESMR‐L was not. Therefore, ESMR‐L is considered to be a more efficient treatment method than ESD for small rectal NETs, especially for less‐experienced endoscopists.

## CONFLICT OF INTEREST

None.

## FUNDING INFORMATION

This work was supported by the Kumamoto University Hospital Research Revitalization Project.

## Supporting information


**Table S1** The clinical outcomes of each of the 11 institutions
**Table S2** Subgroup analysis to evaluate the impact of less‐experienced endoscopists on treatment outcomes using linear or logistic regression model adjusted by propensity score
