# Supplementary material for: Comparison of endoscopic submucosal resection with ligation and endoscopic submucosal dissection for small rectal neuroendocrine tumors: A multicenter retrospective study
Source: DEN Open. 2022 Sep 15;3(1):e163. doi: 10.1002/deo2.163 (PMC9478042; doi:10.1002/deo2.163)
Supplement: Supplementary file 1 — Table S1 The clinical outcomes of each of the 11 institutions Table S2 Subgroup analysis to evaluate the impact of less‐experienced endoscopists on treatment outcomes using linear or logistic regression model adjusted by propensity score [file DEO2-3-e163-s001.docx]

**Supplemental table 1.** The clinical outcomes of each 11 institutions

| Institutions | No. of  Patients | Operator, no. | R0 resection rate, % | Complication, % |
| --- | --- | --- | --- | --- |
|  |  | Experienced / less-experienced |  |  |
| A | 53 | 51 / 2 | 98.1 | 3.8 |
| B | 42 | 20 / 22 | 88.1 | 7.1 |
| C | 31 | 28 / 3 | 83.9 | 6.5 |
| D | 24 | 19 / 5 | 91.7 | 0 |
| E | 18 | 7 / 11 | 94.4 | 0 |
| F | 15 | 13 / 2 | 80.0 | 0 |
| G | 8 | 6 / 2 | 100 | 12.5 |
| H | 8 | 4 / 4 | 100 | 0 |
| I | 3 | 2 / 1 | 100 | 0 |
| J | 2 | 1 / 1 | 50.0 | 0 |
| K | 1 | 1 / 0 | 100 | 0 |

**Supplemental table 2.** Subgroup analysis to evaluate the impact of less-experienced endoscopists on treatment outcomes using linear or logistic regression model adjusted by propensity score.

| ER method | ESMR-L | | ESD | |
| --- | --- | --- | --- | --- |
|  | The ratio | P value | The ratio | P value |
|  | (95% CI) |  | (95% CI) |  |
| Procedure time | 1.14 | 0.205 | 1.42 | 0.009 |
|  | (0.92- 1.40) |  | (1.12- 1.80) |  |
| R0 resection rate | 3.94 | 0.282 | 0.75 | 0.716 |
|  | (0.32- 47.96) |  | (0.16- 3.53) |  |

ESMR-L, endoscopic submucosal resection with ligation; ESD, endoscopic submucosal resection; IQR, Inter Quartile Range; The ratio, the technical outcome (procedure time, R0 resection rate) of less-experienced compared with experienced endoscopists.
